# Hepatitis C, Innate Immunity and Alcohol: Friends or Foes?

**DOI:** 10.3390/biom5010076

**Published:** 2015-02-05

**Authors:** Natalia A. Osna, Murali Ganesan, Kusum K. Kharbanda

**Affiliations:** 1 Research Service, Veterans Affairs Nebraska-Western Iowa Health Care System, 4101 Woolworth Ave, Omaha, NE 68105, USA; E-Mails: murali.ganesan@unmc.edu (M.G.); kkharbanda@unmc.edu (K.K.K.); 2 Department of Internal Medicine, University of Nebraska Medical Center, Omaha, NE 68105, USA

**Keywords:** HCV, ethanol, HCV RNA, innate immunity, toll-like receptors, interferon signaling, hepatocytes, dendritic cells

## Abstract

Hepatitis C and alcohol are the most widespread causes of liver disease worldwide. Approximately 80% of patients with a history of hepatitis C and alcohol abuse develop chronic liver injury. Alcohol consumption in hepatitis C virus (HCV)-infected patients exacerbates liver disease leading to rapid progression of fibrosis, cirrhosis and even hepatocellular carcinoma. Hepatocytes are the main sites of HCV-infection and ethanol metabolism, both of which generate oxidative stress. Oxidative stress levels affect HCV replication and innate immunity, resulting in a greater susceptibility for HCV-infection and virus spread in the alcoholic patients. In this review paper, we analyze the effects of ethanol metabolism and other factors on HCV replication. In addition, we illustrate the mechanisms of how HCV hijacks innate immunity and how ethanol exposure regulates this process. We also clarify the effects of HCV and ethanol metabolism on interferon signaling—a crucial point for activation of anti-viral genes to protect cells from virus—and the role that HCV- and ethanol-induced impairments play in adaptive immunity which is necessary for recognition of virally-infected hepatocytes. In conclusion, ethanol exposure potentiates the suppressive effects of HCV on innate immunity, which activates viral spread in the liver and finally, leads to impairments in adaptive immunity. The dysregulation of immune response results in impaired elimination of HCV-infected cells, viral persistence, progressive liver damage and establishment of chronic infection that worsens the outcomes of chronic hepatitis C in alcoholic patients.

## 1. Introduction

Hepatitis C and alcohol are the most widespread causes of liver disease worldwide, and approximately 80%–90% of alcohol-abused HCV patients develop chronic liver injury [1]. The prevalence of hepatitis C is seven to 10-fold higher in alcoholics than it is in the general population, and among alcoholics, the prevalence of hepatitis C is higher in people with advanced liver disease [2]. Alcohol consumption in hepatitis C virus (HCV)-infected patients exacerbates liver disease leading to rapid progression to fibrosis, cirrhosis and even hepatocellular carcinoma [3]. In fact, in some studies, liver cirrhosis is reported as an outcome of HCV-infection in 16.9% of cases and as an outcome of alcoholic liver disease in 8.4% of cases, while in HCV^+^ alcoholics it rises to 27.2% [4]. These effects are related to the dose of consumed alcohol since heavy alcohol intake contributes to fibrosis in patients with HCV independent of other predictors [5]. Heavy alcohol (50 grams/day or more) intake is equivalent to five or more drinks per day. An “average” drink corresponds to one 12-oz beer, 5 oz of wine, or one 1.25 oz shot of hard liquor—each containing approximately 10 grams of ethanol. Heavy drinkers become almost insensitive to interferon treatment [6].

Liver is a primary site of ethanol metabolism. Ethanol is metabolized to acetaldehyde by two major enzyme systems, alcohol dehydrogenase (ADH) and microsomal ethanol-oxidizing system (MEOS), cytochrome P4502E1 (CYP2E1). CYP2E1, in addition, generates both stable, nontoxic and highly unstable (*i.e.*, reactive) harmful molecules, called free radicals. Free radicals include superoxide (O_2_), hydrogen peroxide (H_2_O_2_), and hydroxyl radicals (OH^.^). An excess of oxygen radicals causes oxidative stress, thereby attacking vital cell components, fat and protein constituents of the cell wall and the nucleus (DNA). These reactive oxygen species (ROS) may contribute to the development of fatty liver and fibrosis [7].

HCV proteins are able to induce oxidative stress. It has been shown that by binding to the mitochondrial outer membrane, HCV core protein facilitate the influx of calcium into the mitochondria, thereby stimulating electron transport and ROS production [3,8]. Non-structural HCV proteins, NS5A and NS3 also induce ROS [9,10]. In addition, HCV proteins deplete cell protection by decreasing glutathione levels [8,11] and finally, increase liver cell death by apoptosis in CYP2E1+ cells [12]. Increased oxidative stress seems to be the dominant mechanism for this synergism between alcohol and the HCV [13]. Alcohol also potentiates the quasi-species complexity of HCV in hypervariable region 1 [14,15] by up-regulating the virus-induced oxidative/nitrosative stress to modulate the basal mutation rate of HCV [16]. However, the exact molecular mechanisms of alcohol-induced aggravation of HCV-infection remain uncertain.

In this review, we will focus on the effects of alcohol on HCV replication and on the effects of HCV by itself and alcohol in breaking the host defense mechanism to ultimately suppress the protection of liver cells from the virus. Unfortunately, as becomes clear from this review, information about the combined effects of HCV and alcohol is very limited, and this area of investigation, especially in the context of ethanol metabolism, should be expended.

## 2. Ethanol and HCV Replication

Several studies confirm the hypothesis that alcohol acts as a cofactor in promoting HCV RNA expression. However, the reported effects of ethanol depend on the experimental models used that have created some controversy. McCartney *et al.* reported no increase in viral replication in ethanol-non-metabolizing (CYP2E1-negative) cells but observed an increase in CYP2E1^+^ cells [17]. This increase was attributed to CYP2E1-dependent ROS formation as it was prevented by the effects of antioxidants [17]. Conversely, studies from another group [18,19] demonstrated that ROS formation and lipid peroxidation suppressed while acetaldehyde (low dose), acetone and acetate enhanced HCV replication. In addition, elevated NADH/NAD ratio, up-regulated total cholesterol content and inhibited β-oxidation, together increased HCV RNA levels [18]. Ye *et al.* using human hepatocytes conducted studies on HCV replication [1]. However, their studies were unable to shed any light on the effects of ethanol metabolites since HCV infection was measured after six days in culture, while hepatocytes lose expression of the ethanol-metabolizing enzymes due to the rapid de-differentiation in 24–48 h [20]. Interestingly, some studies reported enhanced HCV replication by ethanol in ethanol-non-metabolizing Huh7.5 or Huh7 cells [1,21,22]. In this case, the effects of ethanol were explained by the elevation of HSP90 and GW182 linked to miR-122 biogenesis [22]. Another miR-122-mediated mechanism suggests that alcohol increases HCV RNA replication by regulating miR-122 and Cyclin G1 [21]. The up-regulating effects of siRNAs (miR-122) on HCV replication indeed have been shown before [23]. In our studies where extracellular acetaldehyde-generating system was used to treat JFH1-infected Huh7.5 cells, we observed the reduction in HCV RNA, which could not be reversed by CYP2E1 transfection [24]. Importantly, using the model of Scid Alb-uPA HCV-infected chimeric mice with humanized liver, we observed longer persistence of HCV in mice fed ethanol in water for five weeks compared with control mice [25]. These results indicated that alcohol exposure may prevent the resolution of HCV-infection, thereby promoting the chronic course of disease. However, HCV RNA levels obtained in alcohol-consuming and non-consuming HCV-patient in clinical trials are also not consistent. While some studies demonstrated higher levels of HCV RNA in blood of alcohol-consuming patients [26,27], a more detailed analysis of the correlations of HCV replication and drinking status showed no link between these two factors [28]. Thus, clinically relevant potentiation of HCV-infection severity by alcohol cannot be exclusively explained by the effects of ethanol on HCV replication.

## 3. HCV-Impaired Innate Immunity

**Figure 1:**
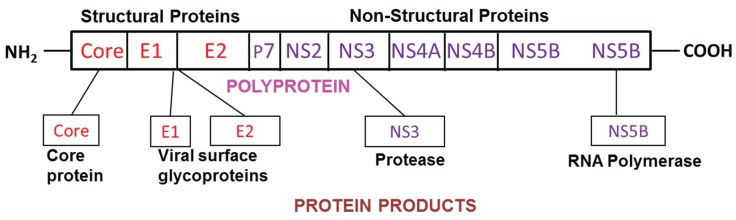
HCV genome.

A member of Flaviviridae, HCV is a positive stranded RNA virus. Its genome has approximately 9600 nucleotides containing a large open reading frame coding for a polyprotein, which, in turn, is processed into 10 separate proteins. Among these, core (nucleocapside protein), NS3 (helicase/protease), NS5a, and NS5b (RNA polymerase) proteins have been implicated in HCV-related tissue damage and carcinogenesis. HCV genome is presented in Figure 1.

Due to the exclusive ability to evade immune response, HCV became a champion in hijacking innate immunity defense. Usually, when cells are infected with a virus, the recognition of a pathogen-associated molecular pattern (PAMP) is sensed by pattern recognition receptors (PRR).

### 3.1. HCV and Viral dsRNA Recognition

**Figure 2:**
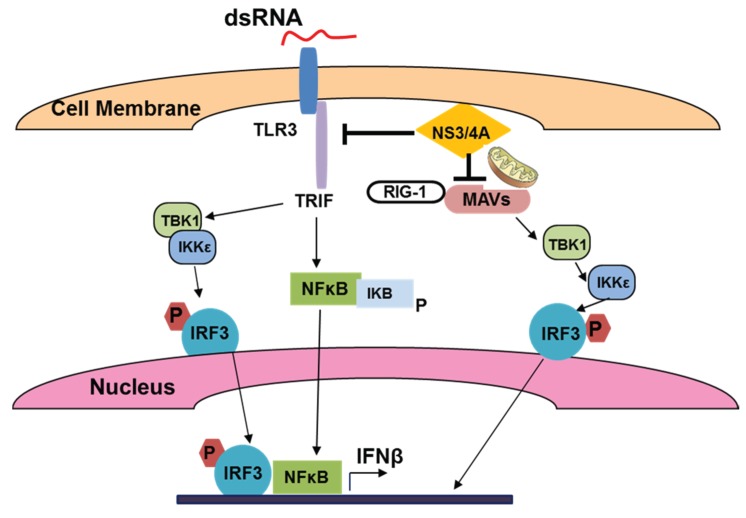
Sensing of viral (HCV) dsRNA by TLR3 and activation of IFNβ production.

For viral dsRNA recognition, the major PRRs include intracellular retinoic-inducible gene 1 (RIG1) and melanoma differentiation associated gene 5 (MDA5), as well as Toll-like receptor 3 (TLR 3) expressed on the membrane of liver cells. Interestingly, lack of TLR3 and a mutation in RIG-1 in Huh 7.5 cells make them highly permissive to HCV-replication [29] that allows successful usage of these cells for *in vitro* HCV studies. After infection, the *C*-terminal domain of RIG1/MDA5 undergoes a conformational change, driving a response through the mitochondrial antiviral signaling protein (MAVS), which serves as an adaptor in IFN signaling and is localized both at peroxisomes and mitochondria [30,31]. Peroxisomal MAVS activates early interferon sensitive genes (ISG) by induction of interferon regulated factors 1 and 3 (IRF1 and IRF3), in the absence of interferon, while mitochondrial MAVS activates TBK1 and IKK kinases to phosphorylate IRF3, leading to IFN induction and NFκB activation (reviewed by [32]), summarized in Figure 2.

The mitochondrial-associated membrane provides the link between mitochondria and endoplasmic reticulum (ER), and is implicated in inflammasome signaling [31]. In addition to RIG1 signaling, viral dsRNA is sensed by TLR3 in late endosomes or lysosomes, and thus, this signaling can be abrogated by acidification/autophagy inhibitors, bafilomycin A1 and cloroquine [33]. TLR3 signaling recruits the TIR (Toll-interleukin receptor)-domain containing adapter TRIF (TLR domain-containing adapter inducing IFNβ) that activates IKK complex, NFκB and mitogen-activated protein kinase (MAPK) and up-regulates IFNβ through TLR3 activation [34,35].

### 3.2. HCV and ssRNA Recognition

ssRNA recognition requires TLR7 and TLR8, which are also located in late endosome and requires acidification for signaling. TLR7 is expressed predominantly on plasmocytoid dendritic cells (pDC), but TLR7 mRNA is detected in hepatocytes. Triggering of TLR7 induces IFNα production [36,37]. TLR8 receptors are expressed in myeloid dendritic cells (mDC) and monocytes and mainly induce NFκB-dependent production of pro-inflammatory cytokines [38]. Since pDC express IRF7 constitutively, they produce Type 1 IFNs very rapidly, without involvement of IFN-induced autocrine/paracrine signaling [39].

### 3.3. HCV, TLR Expression and Innate Immunity in Hepatocytes

HCV uses various mechanisms to circumvent the IFN response by blocking downstream signaling via changes in repertoire of IFN Stimulatory Genes (ISGs) [40]. Paradoxically, high steady-state activation of ISGs in HCV patients not only impedes the protection from chronic infection, but even correlates with the poor response to interferon treatment [41]. This occurs because HCV has an effective strategy to resist innate immunity response. HCV proteins regulate expression of TLRs on the surface of infected cells (hepatocytes) and non-parenchymal and/or immune cells that are interacting with infected hepatocytes. When TLR expression has been examined in HepG2 cells transfected with entire or parts (core-NS3, NS3-NS5B) of the HCV open reading frame, TLR3 expression was suppressed only by entire gene transfectant, indicating that several HCV antigens control TLR3 activation [42]. The most prominent suppressor of TLR3-mediated signaling is HCV protease NS3/4A. As shown, in hepatocytes, NS3/4A breaks the host defense mechanism by blocking TLR3 and RIG1-mediated signaling by disrupting the adaptor proteins, MAVS and Toll/IL-1 receptor domain-containing adapter inducing IFN-β (TRIF) necessary for early ISG activation [43,44]. While core protein stimulates the TLR2 pathway that assists the virus to evade the innate immune system, NS3/4A disrupts TLR3 and RIG-1 signaling pathways by cleaving TRIF and MAVS, which leads to persistence of infection [45]. In addition, IFNβ production in HepG2 cells co-transfected with various HCV plasmids was even enhanced by NS5B (viral RNA polymerase) via activation of Interferon Regulator Factor 3 (IRF3), but was blocked by NS4A, NS4B, and NS5A [46]. Expression and function of other TLRs (like TLR7 that senses single-strand RNA) are also impaired by HCV infection in liver cells [47].

### 3.4. HCV, TLR Expression and Innate Immunity in Immune Cells

In dendritic cells of chronic hepatitis C patients, TLR3 expression is not changed, while TLR2 expression is reduced [48]. In addition, IFNα production capacity of blood plasmocytoid dendritic cells (PDC) as well as allostimulatory capacity of myeloid dendritic cells (MDC) were significantly lower in patients with chronic HCV infection compared to normal controls, and TLR2 appeared to be essential for the recognition of HCV core and NS3 proteins by innate immune cells [49,50]. Hoffman *et al.* confirmed TLR2 indeed recognizes HCV proteins (namely, core protein), but not the viral particle [51]. Since HCV core and NS3 proteins induced TNF-alpha and IL-10 production in human monocyte-derived macrophages, and this induction was impaired by TLR2, TLR1, and TLR6 knockdown, it was suggested that TLR2 may use TLR1 and TLR6 co-receptors for HCV core- and NS3-mediated activation of macrophages and subsequent innate immunity in humans [52]. Even if dendritic cells are not infected with HCV, the virus inhibits PDCs, but not MDC and monocytoid-derived DCs, via direct interaction of released viral particles with cells [53]. As revealed in a recent study, circulating HCV core protein interferes with TLR signaling in pDCs to suppress IFN production due to impairment of Interferon Regulated Factor 7 (IRF7) expression and STAT1 phosphorylation [54]. Importantly, it has been shown that TLR3- and TLR4-stimulated non-parenchymal cells, in turn, are able to regulate HCV replication in infected cells through the production of IFN-β, which partly explains the source of high levels of ISG expression in HCV infected liver [55]. Moreover, all HCV-related proteins studied markedly enhance the production of the pro-inflammatory cytokine, TNF-α, by Kupffer cells which wassimilarto LPS-mediated stimulation; this production in response to NS3 was significantly blunted by neutralization antibodies against the TLR4 [56]. HCV enters the KC via phagocytic uptake, with no development of the productive infection, and this leads to IFNβ-mediated activation of innate immunity through the RIG1/MAVS pathway and inflammatory signaling through the TLR7/myD88 and NLRP3 inflammasome pathway [57]. Importantly, activation of macrophages/KC with a panel of TLR agonists induce soluble mediators that interact with HCV and promote infection spread by increasing permissiveness of neighboring hepatocytes to HCV. Thus, TNFα-induced relocation of tight junction proteins (occludin, claudin 1), that serve as the receptors for HCV entry, increase their lateral cell localization, which enables the entrance of HCV into TNFα-pre-sensitized hepatocytes [58]. However, nothing is known about the effects of ethanol on HCV entry.

Cellular immunity, including natural killer cell activation and antigen-specific CD8 T-cell proliferation, occurs following innate immune activation in response to HCV, but is often ineffective for eradication of HCV [59]. The infected hepatocytes (through the discontinuous, fenestrated endothelial cell lining in the liver) have a direct contact with immune cells that impairs dendritic cells and NK cell functions, causing reduced innate immunity and failure to generate effective T cell responses to clear HCV infection [60]. NK cells play a significant role in anti-HCV innate immunity [61]. However, NS5A also down-regulates the expression of NKG2D on another important innate immunity player, natural killer cells (NK cells), via the TLR4 pathway and impairs the functional ability of these cells [45]. All of these evasion mechanisms make HCV one of the most efficient viruses to hijack the host’s immunity, leading to unbeatable survival of the virus in infected cells [45]. To further survive in hepatocytes, HCV tries to prevent apoptotic cell death. To this end, NS5A inhibits the expression of numerous genes encoding for molecules involved in TLR4 signaling (CD14, MD-2, myeloid differentiation primary response gene 88, IRF3, and NF-κB), thereby disrupting LPS-induced TLR4-mediated apoptosis in human hepatocytes [62], which finally affects HCV-infection pathogenesis.

### 3.5. Interferons and Their Role in Innate Immunity

**Figure 3:**
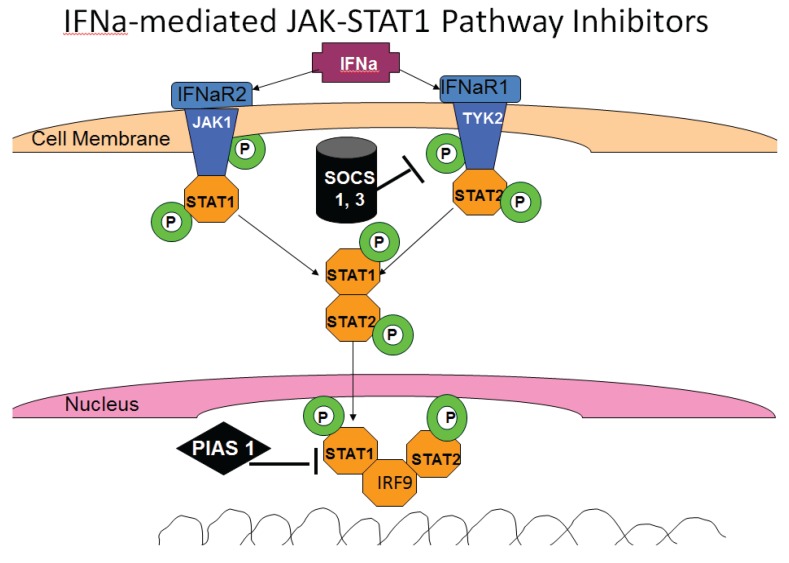
IFN-induced signaling through the JAK-STAT pathway.

Interferon production is an important component of innate immunity. IFNs by themselves do not provide direct antiviral activity, but they activate interferon-sensitive genes (ISGs) that, in turn, induce proteins/signal transduction factors possessing anti-viral properties. In addition, IFNs play a role in activation of effector immune cells and provide a link between innate and adaptive immunities. IFNs also regulate pro-inflammatory cytokine and chemokine release, induce cell immune cell maturation, slow down cell proliferation and promote apoptosis [61]. Depending on the receptors that cells utilize for activation by interferons, type 1, type 2 and type 3 IFNs are identified. The **type 1 IFNs** include interferon alpha and interferon beta (IFNα and IFNβ). They are recognized by IFNR1 and IFNR2 that are expressed in all cell types. Triggering of IFN receptors induces the phosphorylation of intracellular Janus kinases, JAK1 and TYK2, which create the anchoring sites for transcription factors, STAT1 and STAT2. Phosphorylation of STAT2 is followed by phosphorylation of STAT1, dimerization, recruitment of IFN-Regulatory Factor 9 (IRF9) and translocation of this trimeric structure to nucleus to activate ISGs of IFN-Stimulated Response Element (ISRE), responsible for anti-viral, anti-microbial and tumor responses [63], summarized in Figure 3. Alternatively, STAT1 dimerizes and after translocation to nucleus, activate genes of Gamma IFN-activated Site (GAS) area [64,65,66].

**Type 2 IFN** includes Interferon gamma (IFNγ), which is produced by T-lymphocytes and NK cells and signals via IFNR1 and IFNR2. Triggering these receptors induces activation of Janus kinases, JAK1 and JAK2 followed by STAT1 phosphorylation, dimerization and attachment to GAS area of genes mainly responsible for activation of immune response and indirectly, for activation of anti-viral/microbial response [67].

**Type 3 IFNs** (IL-29, IL28A and IL28B, or ƛ1, 2, 3, respectively) activate the JAK-STAT pathway similar to type I IFNs, but using IL28R1 and IL10R2, mainly on epithelial cells, dendritic cells and hepatocytes. Type 3 IFNs induce the same set of genes as IFNs type 1, but the kinetics is different [67]. These IFNs are anti-viral, but structurally they are close to IL-10 family cytokines [68].

### 3.6. HCV and IFN Signaling

In HCV infection, even if IFN is produced by non-parenchymal cells that are not infected, the activation of antiviral properties in infected hepatocytes requires the potent IFN signaling through the JAK-STAT1 pathway to achieve the protection from virus/viral clearance. However, IFN signaling in hepatocytes is not effective because HCV interferes with IFN signaling by preventing activation of “late” ISGs and blocks STAT1, STAT2, IRF9 and JAK-STAT pathways [40]. The virus suppresses STAT1 signaling by degrading ubiquitylated STAT1 [69] as well as IFNα-activated STAT3 [70]. NS5A protein was shown to decrease STAT1 phosphorylation in liver cells [71]. In addition, suppression of STAT1 phosphorylation by HCV is related to HCV-mediated induction of Suppressors of Cytokine Signaling 3 (SOCS3), which block STAT1 phosphorylation (Figure 3) [72]. Furthermore, at the downstream level of the JAK-STAT1 signaling, HCV induces protein phosphatase, PP2A, which blocks arginine protein methyltransferase 1 (PRMT1)-mediated methylation of STAT1 necessary for attachment of phosphorylated STAT1 to DNA [73,74,75]. Impaired STAT1 methylation may cause enhanced complex formation between protein inhibitor of activated STAT1 (PIAS1) and STAT1, which competes with STAT1 binding to DNA (Figure 3). Numerous reports from cell-culture studies suggest that HCV causes PP2A up-regulation by inducing an endoplasmic reticulum stress response [76], thereby reducing the ability of cells to respond to IFN. The impairment of IFN-induced signaling through the Jak-STAT1 pathway also serves as a negative regulator of proteasome/immunoproteasome activation, which is modulated through this pathway. In monocytes/macrophages, TLR-dependent production of IL-12 is negatively regulated by expression of the receptor Programmed death-1 (PD-1), which suppresses STAT1 phosphorylation during chronic HCV infection [77].

### 3.7. HCV and ISG Activation

HCV also affects the functions of some ISGs, thereby protecting the virus instead of the host. As an example, protein kinase R (PKR), via its ability to control translation, through phosphorylation of the alpha subunit of eukaryotic initiation factor 2 (eIF2α), was initially recognized as a regulator of the antiviral action of IFN. PKR regulates several signaling pathways (NF-κB, p38MAPK and insulin pathways) either as an adapter protein or via its kinase activity that additionally can block HCV RNA replication. In HCV, PKR interacts with HCV core protein and NS5A or E2 [78,79]. Furthermore, PKR recently was characterized as a pro-HCV agent, both as an adapter protein and as an eIF2α-kinase, which acts in cooperation with the di-ubiquitin-like protein, ISG15 [80]. Normally, for initiation of innate immunity activating signaling, RIG1 and MAVS are to be ubiquitylated by the E3 ligases, Trim25 [81,82]. However, as an alternative to ubiquitylation, HCV induces ISGylation of RIG1 by a small ubiquitin-like modifier, ISG15, which prevents RIG1 ubiquitylation [83], thereby blocking RIG1-induced signaling. Since protective innate immunity in infected cells is not activated, it may increase permissiveness of HCV infection in hepatocytes [59]. As a result, HCV is eliminated during the acute phase of the infection in about 30% of patients, while it persists for decades in the remaining 70% of patients [84].

## 4. Ethanol-Regulated HCV Innate Immunity

The prevalence of HCV is significantly higher among alcoholics than in the general population. For example, while the HCV positive rate in the general population of the U.S. is roughly 1%, it is 16% for alcoholics and nearly 30% for alcoholics with liver diseases [85]. However, the exact knowledge about the combined effects of HCV and ethanol on innate immunity is very limited.

### 4.1. Ethanol, IRFs and TLRs

It appeared that some innate immunity factors, which are supposed to protect cells from the virus, may induce the unwanted changes in response to ethanol depending on the cell type affected by ethanol exposure. As an example, the whole-body interferon regulatory factor 3 knockout (IRF3-KO) mice were protected from alcohol-induced liver injury, steatosis, and inflammation, while in wild-type or bone marrow-specific IRF3-KO mice, deficiency of IRF3 only in parenchymal cells aggravated alcohol-induced liver injury and was associated with increased pro-inflammatory cytokines, lower anti-inflammatory cytokine interleukin 10 (IL-10), and lower Type I IFNs compared to wild-type mice [86]. Further, ethanol disrupted activation through TLRs that are crucial for innate immunity induction. To this end, suppression of TLR3 signaling in liver cells by ethanol was supported by some studies [87,88]. Additionally, TLR3 activation ameliorates alcoholic liver injury via the stimulation of IL-10 production in hepatic stellate (HSCs) and Kupffer cells [89]. Since HCV also blocks TLR3 signaling, there is a possibility that ethanol would provide a synergistic suppressive effect on innate immunity, but these studies have not been done yet. In addition to TLR3 studies, modulation of type 1 IFN production in human macrophages was achieved through triggering of both TLR8 and TLR4 and was attenuated by both acute and chronic ethanol exposure [90]. Chronic ethanol consumption activates other TLRs, TLR1 and 2, 6, 9 that increase TNFα-induced response to LPS in mice [91].

Human monocytes exposed to ethanol become hypersensitive to LPS via the activation of mitogen-activated protein kinase through TLR 4 signaling, followed by activation of NF-kB, AP-1 and ERK [92]. Although most of these studies were not conducted in the presence of HCV/HCV proteins, they still provide a clue of how ethanol may be potentiating the effects of HCV in promoting liver injury. In addition, several possible mechanisms of HCV-ethanol interactions were reviewed [93]. These include enhanced secretion of TNF, IL-1, IL-6 and IL-8, induction of apoptosis via activation of TRADD/FADD signaling and depletion of mitochondrial glutathione as well as other protective systems leading to increased sensitivity of liver cells to TNF.

### 4.2. Ethanol and TLR-Related Outcomes of HCV-Infection

The outcomes of HCV-infection are severely worsened by ethanol exposure. As revealed from another study, the combined effects of HCV proteins and alcohol tremendously enhance a chance for outcomes to hepatocellular carcinoma (HCC). Thus, a daily intake of 80 g alcohol alone increases HCC risk five-fold, while the presence of HCV alone increases it 20-fold; the combination of HCV and alcohol increases the risk for HCC over 100-fold [94]. HCV-infection (NS5A protein) up-regulates TLR4 expression and pro-inflammatory cytokine production, making hepatocytes very sensitive to ethanol-induced LPS effects. Finally, NS5A-expressing mice fed alcohol for 12 months, develop liver tumors in a manner dependent on NS5A-mediated induction of TLR4 [95]. Ethanol-enhanced TLR4 signaling in these mice, in turn, up-regulates the stem cell marker, Nanog, and is required for TLR4-dependent liver oncogenesis [95]. The incidence of spontaneous HCC induction was two-fold increased by chronic alcohol intake in the HCV core transgenic mice [96].

The reason for alcohol-mediated increase in HCC is not only due to the impairment of immune responses, but also due to the fact that ethanol metabolites, acetaldehyde and ROS, have a role in alcohol-related carcinogenesis because they can form DNA adducts that are prone to mutagenesis. These adducts interfere with methylation, synthesis and repair of DNA, thereby increasing HCC susceptibility [97].

**Figure 4:**
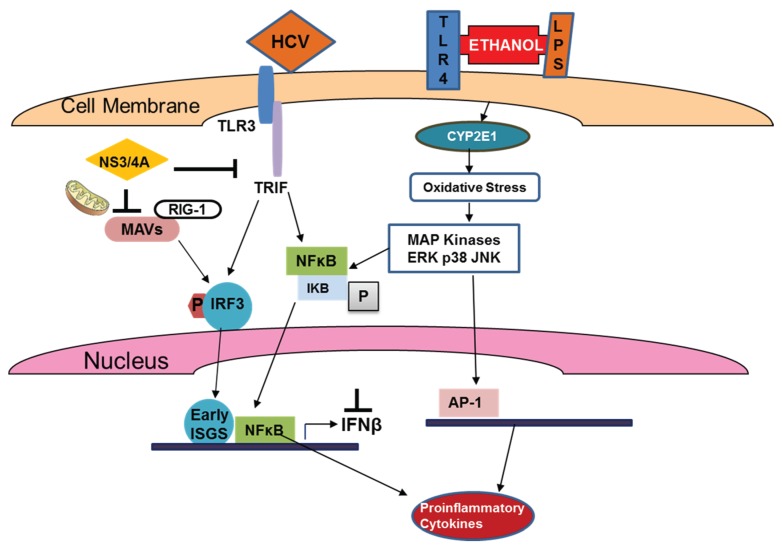
Effects of HCV and ethanol on activation of early ISGs and genes responsible for production of pro-inflammatory cytokines.

The combined effects of HCV and ethanol/LPS are summarized in Figure 4. It shows that while HCV blocks IRF3 phosphorylation and subsequent activation of early ISGs, ethanol and LPS still induce stress response to activated pro-inflammatory cytokine production via multiple signaling pathways.

### 4.3. Ethanol, HCV and IFN Signaling

Most of the published data are related to ethanol-induced defects in IFN-activated signaling through the JAK-STAT1 pathway. Acute ethanol exposure to HCV-expressing Huh7 cells induced STAT1 serine phosphorylation partially mediated by the p38 MAPK pathway. In contrast, when combined with exogenous IFN-alpha, ethanol inhibited the antiviral actions of IFN against HCV replication by inhibiting IFN-induced STAT1 tyrosine phosphorylation [98]. In addition, acute ethanol exposure significantly potentiated IFN-β or IFN-γ-induced activation of p42/44 MAPK, and caused marked activation of protein kinase C (PKC). Inhibition of PKC partially antagonized ethanol attenuation of IFN-induced STAT1 activation, suggesting that PKC may be involved in the regulation of STAT1 activation by acute ethanol treatment [99]. Also, in HCV-infected cells, alcohol inhibited the expression of the IFN regulatory factors (IRF-5 and IRF-7), and signal transducer and activator of transcription (STAT-1 and STAT-2), and induced suppressors of cytokine signaling (SOCS-2 and SOCS-3), the negative regulators in type I IFN signaling pathway [1]. However, these experiments were not done using the cell lines or conditions that allowed for ethanol metabolism. As revealed from our previous studies, ethanol metabolism through CYP2E1 and alcohol dehydrogenase is required for suppressive effects of ethanol on IFN signaling [17,100,101]. Furthermore, experiments on CYP2E1-expressing HCV replicon cells demonstrated that ethanol metabolism, but not ethanol-induced reduction in STAT1 and STAT2 phosphorylation on both tyrosine and serine residues, correlated with a decreased ISRE-promoter activity [17]. Additionally, miRNAs in exosomes may play a role in a synergism between HCV and alcohol. In alcoholic liver disease as well as in inflammatory liver injury, serum/plasma miR-122 and miR-155 were predominantly associated with the exosome-rich fraction [102], and HCV RNA is also known to be up-regulated by miR-122 [23]. Taking into account the data thatexosomesmediatethe cell-to-cell transmission of IFNα-induced antiviral activity [103], it can be assumed that both ethanol and HCV may affect cross-talks between various cell types in the liver and subsequent anti-viral protection.

### 4.4. Ethanol and Adaptive Immune Response

Alcohol consumption in HCV patients affects adaptive immune response by affecting immune cells, namely DCs that are critically important for the generation of adaptive immune response. Specifically, DCs that differentiate *in vivo* during chronic alcohol feeding may have intrinsic functional defects. This could partially explain the depressed cytotoxic T cell lymphocyte (CTL) activity previously observed after DNA-based immunization by HCV core and NS5 as the immunogens [104,105]. Thus, both ethanol and HCV synergize to impair antigen presentation by “professional” antigen presenters. In addition to HCV and ethanol-impaired function of professional antigen-presenting cells that activate specific effector immune cells, there is a reduced ability of HCV-expressing hepatocytes to process “foreign” proteins for presentation in the context of HCV class I in ethanol-fed HCV core transgenic mice [106]. This occurs due to suppression of the major intracellular protein-degrading enzyme—the proteasome—which trims the peptides for MHC class I-restricted presentation for recognition by cytotoxic T-lymphocytes and removal of the infected hepatocytes. Ethanol metabolism in liver cells indeed, is responsible for proteasome inhibition followed by impairment of antigen-processing machinery [107,108]. This impairment of proteasome function is regulated by multiple mechanisms: oxidative stress, methylation status of cells and HCV-proteasome direct interactions [11,109]. Importantly, even if HCV proteins and ethanol exposure alone do not induce sufficient changes to suppress proteasome activity, the combination of these factors does so by achieving the high level of oxidative stress [11].

### 4.5. Treatment of HCV-Infection in Alcohol-Abused Patients

Alcohol also reduces responsiveness of HCV patients to anti-viral treatments, and only 7% of heavy drinking HCV patients respond to interferon (IFN) therapy. Currently, it is not clear whether alcohol abuse would affect the outcomes of direct anti-viral agent (DAA)-treatment, including Sofosbuvir. It is predicted that with increasing mutations in NS5B region, the resistance to Sofosbuvir in HCV patients may increase [110]. Furthermore, the responsiveness rate in alcohol abused hepatitis C patients might be even lower than in the general population since chronic alcohol exposure increases the rate of the quasi-species complexity of HCV, making virus less sensitive to DAA. In addition, the success of therapy with DAA is also based on the restoration of innate immunity (and activation of IFN-sensitive anti-viral genes as the major part of innate immunity) due to blockade of HCV replication. Innate immunity helps to clear the leftovers of virus, which may still persist in small quantities after DAA treatment. However, in alcoholics, the immune response is suppressed not only by HCV, but also by alcohol exposure, and cannot be fully restored by DAA treatment, thereby creating a possibility of new exacerbations or re-infection of patients with HCV. For now, alcohol is listed as a contraindication for the treatment of HCV-infection with DAA, even if the results of trials on this category of patients are not published yet.

## 5. Conclusions

In summary, ethanol exposure potentiates the suppressive effects of HCV on innate immunity, which activates viral spread in the liver and finally, leads to impairments in adaptive immunity. Since HCV is an intracellular virus, the dysregulation of immune response results in impaired elimination of HCV-infected cells, viral persistence, progressive liver damage and establishment of chronic infection that worsens the outcomes of chronic hepatitis C in alcoholic patients.
